# Silencing of Two Insulin Receptor Genes Disrupts Nymph-Adult Transition of Alate Brown Citrus Aphid

**DOI:** 10.3390/ijms18020357

**Published:** 2017-02-21

**Authors:** Bi-Yue Ding, Feng Shang, Qiang Zhang, Ying Xiong, Qun Yang, Jin-Zhi Niu, Guy Smagghe, Jin-Jun Wang

**Affiliations:** 1 Key Laboratory of Entomology and Pest Control Engineering, College of Plant Protection, Southwest University, Chongqing 400715, China; biyueding@163.com (B.-Y.D.); fengshang1994@yahoo.com (F.S.); qiangzhang1996@163.com (Q.Z.); yingxiong333@163.com (Y.X.); qunyang1023@163.com (Q.Y.); jinzhiniu@swu.edu.cn (J.-Z.N.); guy.smagghe@ugent.be (G.S.); 2 Department of Crop Protection, Ghent University, 9000 Ghent, Belgium

**Keywords:** *Aphis* (*Toxoptera*) *citricidus*, insulin receptor genes, RNAi, development

## Abstract

Insulin receptors play key roles in growth, development, and polymorphism in insects. Here, we report two insulin receptor genes (*AcInR1* and *AcInR2*) from the brown citrus aphid, *Aphis* (*Toxoptera*) *citricidus*. Transcriptional analyses showed that *AcInR1* increased during the nymph–adult transition in alate aphids, while *AcInR2* had the highest expression level in second instar nymphs. *AcInR1* is important in aphid development from fourth instar nymphs to adults as verified by dsRNA feeding mediated RNAi. The silencing of *AcInR1* or/and *AcInR2* produced a variety of phenotypes including adults with normal wings, malformed wings, under-developed wings, and aphids failing to develop beyond the nymphal stages. Silencing of *AcInR1* or *AcInR2* alone, and co-silencing of both genes, resulted in 73% or 60%, and 87% of aphids with problems in the transition from nymph to normal adult. The co-silencing of *AcInR1* and *AcInR2* resulted in 62% dead nymphs, but no mortality occurred by silencing of *AcInR1* or *AcInR2* alone. Phenotypes of adults in the ds*InR1* and ds*InR2* were similar. The results demonstrate that *AcInR1* and *AcInR2* are essential for successful nymph–adult transition in alate aphids and show that RNAi methods may be useful for the management of this pest.

## 1. Introduction

Insulin and insulin-like factor signaling (IIS) pathways play important roles in insects such as in body size [1], embryo development [2,3], diapause [4,5], and wing dimorphism [6]. Insulin receptor (InR) is the upstream component of the IIS pathway. InR is a transmembrane receptor that triggers the signal transduction cascade on insulin binding [7]. InR signal transduction primarily passes through the phosphoinositide 3-kinase (PI3K)/protein kinase B (Akt/PKB) pathway [8]. In the PI3K/Akt pathway, InR transmits a signal by the insulin receptor substrate (IRS), resulting in the activation of PI3K. PI3K catalyzes the phosphorylation of phosphatidylinositol-4,5-bisphosphate to phosphatidylinositol-3,4,5-trisphosphate (PIP3). Increased levels of PIP3 are required to activate phosphoinositide-dependent kinase, which in turn activates Akt, resulting in the phosphorylation of many other proteins that affect cell cycle entry, growth and survival [3,9]. In addition to the PI3K/PKB signaling cascade, the target of the rapamycin complex and the Ras/mitogen-activated protein kinase signaling pathways constitute two alternative signaling branches of the IIS pathway [6,10,11,12].

Two types of insect InR receptors, InR1 and InR2, have been identified. In some insects, only one insulin receptor gene occurs. These insects include the Diptera: *Drosophila melanogaster* [13], *Bactrocera dorsalis* [3], and *Aedes aegypti* [14]; Lepidoptera: *Bombyx mori* [15], Blattoidea: *Blattella germanica* [16]; and Coleoptera: *Onthophagus nigriventris* [17]. Both insulin receptor genes have been identified in polymorphic insects, such as *Aphis mellifera* [18], *Solenopsis invicta* [19], *Nilaparvata lugens* [6], *Acyrthosiphon pisum* [2], and the non-polymorphic insect *Tribolium castaneum* [7]. RNA interference (RNAi) methods have been used to investigate the function of insulin receptor genes involved in insect growth [20,21], development and reproduction [7,22], polymorphism [6], and lifespan [23]. For example, the ds*InR*-treated individuals of *B. mori* showed growth inhibition and malformation such as abnormal black body color [22]. In insects with two insulin receptor genes, such as *T. castaneum*, functional diversity occurs. RNAi results in *T. castaneum* indicated that *InR1* and *InR2* have different functions in beetle development and reproduction [7]. Similar functions were also found in *N. lugens* with *InR1* and *InR2* playing opposing roles in controlling the development of the long wing biotype versus the short wing biotype [6].

Aphids are good examples of taxa that have evolved wing dimorphism and reproductive polyphenism [24,25]. Generally, all aphids are born with wing primordia but apterous (wingless) and alate (winged) aphids cannot be distinguished by examining the morphology of first and second instar nymphs [26]. The primordia are degenerating during the second instar nymph–third instar nymph in apterous morphs [27]. In alate morphs, the wing bud develops slowly in each nymphal stadium until the fully formed wing unfolds after the nymph-to-adult molt [26]. Besides wing dimorphism, aphids also have various reproductive modes. In general, offspring are produced by either viviparous parthenogenesis or sexual production. Nymphs undergo four molts during development to become alate or apterous adults [28,29].

The brown citrus aphid, *Aphis* (*Toxoptera*) *citricidus* (Kirkaldy), is an important citrus pest and the main vector of *citrus tristeza virus* (CTV) worldwide. CTV is one of the most destructive and widely distributed diseases of citrus [30,31]. Like other aphid species, *A. citricidus* has alate and apterous morphs. Apterous morphs have high fecundity whereas alates have strong flight muscles and can fly long distances [32]. The life cycle of *A. citricidus* is simpler than that of most aphid species. In most regions, *A. citricidus* is permanently anholocyclic, meaning that there is no sexual cycle in the autumn. All individuals are viviparous parthenogenetic females year round [33]. The strong flight muscles and high fecundity of *A. citricidus* have made control using chemical insecticides difficult. Understanding the molecular regulation of the development process in alate morphs is needed to advance efficient control strategies.

Although the genomes of three species of aphids, including *A. pisum* [29], *Diuraphis noxia* [34], and *Myzus persicae*, have been sequenced, insulin receptor genes have only been characterized in *A. pisum* [2]. In this study, we report (1) two full-length open reading frame (ORF) sequences of the *AcInR1* and *AcInR2* insulin receptors in *A. citricidus*; (2) different expression patterns of *AcInR1* and *AcInR2* at different aphid developmental stages; and (3) evidence for the involvement of *AcInR1* and *AcInR2* in the nymph–adult transition by using dsRNA feeding-mediated RNA interference (RNAi). This study can be useful to analyze the insulin receptors in other aphids. The results would support advanced studies using RNAi technology as a method to manage populations of *A. citricidus*.

## 2. Results

### 2.1. Two Insulin Receptor Genes in A. citricidus

We obtained the open reading frame (ORF) sequences of the *AcInR1* and *AcInR2* insulin receptor genes from *A. citricidus*. *AcInR1* contained an ORF of 4473 bp that encoded 1490 amino acid residues (aa) with a predicted molecular weight of 169.7 kDa and an isoelectric point (pI) of 5.83 (Figure S1), and *AcInR2* contained an ORF of 3963 bp that encoded 1320 aa with a predicted molecular weight of 150.1 kDa and a pI of 5.85 (Figure S2). Although the nucleotide sequence identity was only 43.7% between *AcInR1* and *AcInR2*, AcInR1 and AcInR2 shared highly similar domain architecture: a furin-like cysteine-rich (Fu) region, three fibronectin type 3 (FN3) domains, a single transmembrane (TM) region, a highly conserved tyrosine kinase domain (TyrKc), an “NPXY” motif, and a triple tyrosine cluster (YXXXYY) (Figures S1 and S2).

A Protein Blast (BLASTP) search of the National Center for Biotechnology Information (NCBI) databases (available on: http://www.ncbi.nlm.nih.gov/) and the Aphid Genome Database (available on: http://www.aphidbase.com/) found that the amino acid sequence of AcInR1 shared a similarity of 96%, 95%, and 94% with MpInR1 (*M. persicae*), ApInR1 (*A. pisum*, XP_008185917.1), and DnInR1 (*D. noxia*, XP_015375915.1), whereas AcInR2 shared a 92%, 90%, and 90% similarity with ApInR2 (*A. pisum*, XP_001942660.2), DnInR2 (*D. noxia*, XP_015375915.1), and MpInR2 (*M. persicae*), respectively.

**Figure 1:**
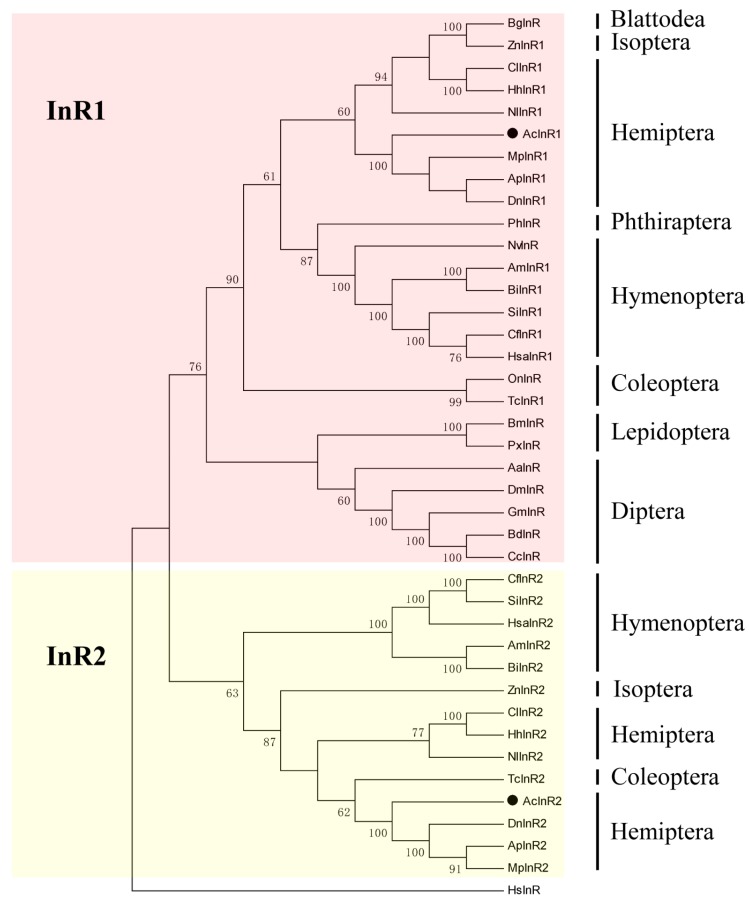
Phylogeny of insect insulin receptors. A phylogenetic tree constructed from amino acid sequences of various insect insulin receptors. The tree was constructed using MEGA 5.05 based on the maximum likelihood (ML) method according to amino acid sequences. Bootstrap support values with 1000 samples are shown on the branches (only those above 50%). Insulin receptors were from *Blattella germanica* (Bg), *Zootermopsis nevadensis* (Zn), *Climex lectularius* (Cl), *Halyomorpha halys* (Hh), *Nilaparvata lugens* (Nl), *Aphis (Toxoptera) citricidus* (Ac), *Myzus persicae* (Mp), *Acyrthosiphon pisum* (Ap), *Diuraphis noxia* (Dn), *Pediculus humanus corporis* (Ph), *Nasonia vitripennis* (Nv), *Apis mellifera* (Am), *Bombus impatiens* (Bi), *Solenopsis invicta* (Si), *Camponotus floridanus* (Cf), *Harpegnathos saltator* (Hsa), *Onthophague nigriventris* (On), *Tribolium castaneum* (Tc), *Bombyx mori* (Bm), *Plutella xylostella* (Px), *Aedes aegypti* (Aa), *Drosophila melanogaster* (Dm), *Glossina morsitans morsitans* (Gm), *Bactrocera dorsalis* (Bd), *Ceratitis capitata* (Cc) *Homo sapiens* (Hs).

To investigate the evolutionary relationship of insect insulin receptors, a phylogenetic analysis based on the full-length amino acid sequences was performed with orthologs from various insect species. InR1 and InR2 separated into two distinct clusters, which indicated that InR2 may play a different role than InR1. Further, InR and InR1 appeared to share a single clade, suggesting that similar physiological functions and evolutionary relatedness exist between InR and InR1 (Figure 1). All aphid insulin receptors seemed to have a common lineage as a high bootstrap value confirmed their phylogeny (Figure 1). InR2 presented in Hymenoptera (ant and bumble bee), Hemipteran (aphids, planthopper, and bugs), Isoptera (dampwood termite), and Coleoptera (red flour beetle) (Figure 1). However, not all species belonging to these groups have two insulin receptors. Only one insulin receptor was found in *Nasonia vitripennis* (Hymenoptera) and *Onthophagus nigriventris* (Coleoptera).

### 2.2. Expression Profiles of AcInR1 and AcInR2 at Different Developmental Stages

**Figure 2:**
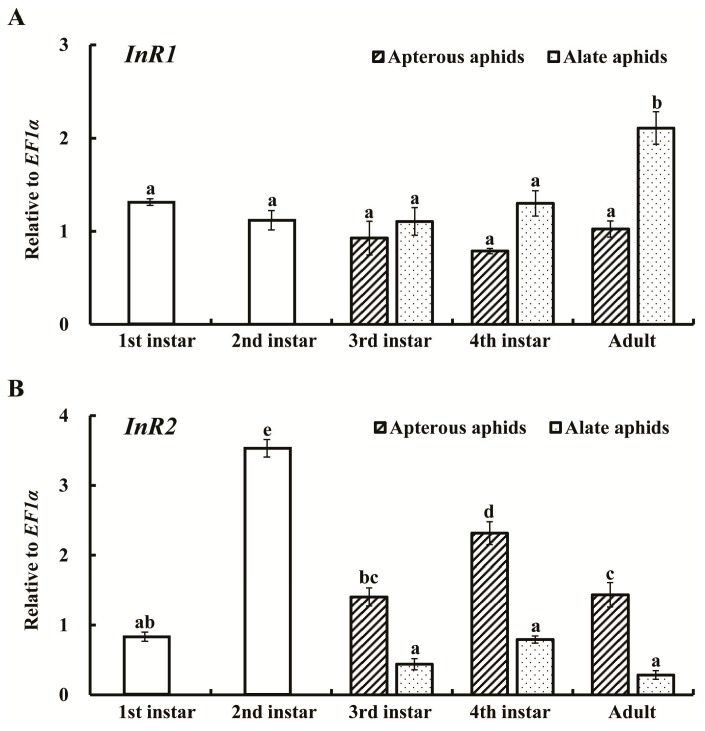
Expression profiles of *AcInR1* (**A**); and *AcInR2* (**B**) at different developmental stages of *Aphis (Toxoptera) citricidus*. The mean (±SE) expression level is based on four biological replicates. Different lowercase letters (a, b, c, d, e) above each bar indicate significant differences among different developmental stages and wing morphs using one-way ANOVA followed by Tukey’s honestly significant difference (HSD) multiple comparison test (*p* < 0.05).

We analyzed the expression patterns of *AcInR1* and *AcInR2* at different developmental stages of *A. citricidus* by quantitative real-time PCR (RT-qPCR). The results showed that *AcInR1* and *AcInR2* were constantly expressed from the first instar nymph to the adult. However, *AcInR1* increased from fourth instar nymphs to alate adults. There was no significant difference among the nymphal stages of alates and also no significant differences were observed among nymphal stages and adults in apterous aphids (Figure 2A). The results indicate that *AcInR1* plays an important role in the development (including wing development) from fourth instar nymphs to alate adults.

*AcInR2* had the highest expression level in second instar nymphs (about 3.4-fold higher than in first instar nymphs). No differences among third instar nymphs, fourth instar nymphs, and adults of alate aphids were observed. In apterous aphids, the expression of *AcInR2* increased with nymphal growth between the third and fourth instars and then decreased in the adults. The expression level of *AcInR2* was higher in apterous aphids compared to alate aphids in third and fourth instar nymphs as well as in adults (Figure 2B). The second instar nymph to third instar nymph was the key period for determining the wing morph (apterous or alate morph) and the fourth instar nymph to adult transition was the important period for wing development in aphids [35]. These results suggest that *AcInR2* may be involved in wing dimorphism and *AcInR1* may play a role in the wing development of *A. citricidus*.

### 2.3. Silencing of AcInR1 and AcInR2 by RNAi Showed Clear Phenotypes

Based on a previously developed method using a plant-stem–mediated dsRNA feeding system [36], the effective and specific silencing of *AcInR1* and *AcInR2* by RNAi was established in *A. citricidus* to further explore the role of these genes in the nymph–adult transition and wing formation. This approach first evaluated the individual silencing of *AcInR1* or *AcInR2* without influencing the expression of the other gene. We also used a mixture of dsRNA to target both *AcInR1* and *AcInR2* in order to evaluate the function of this pathway.

**Figure 3:**
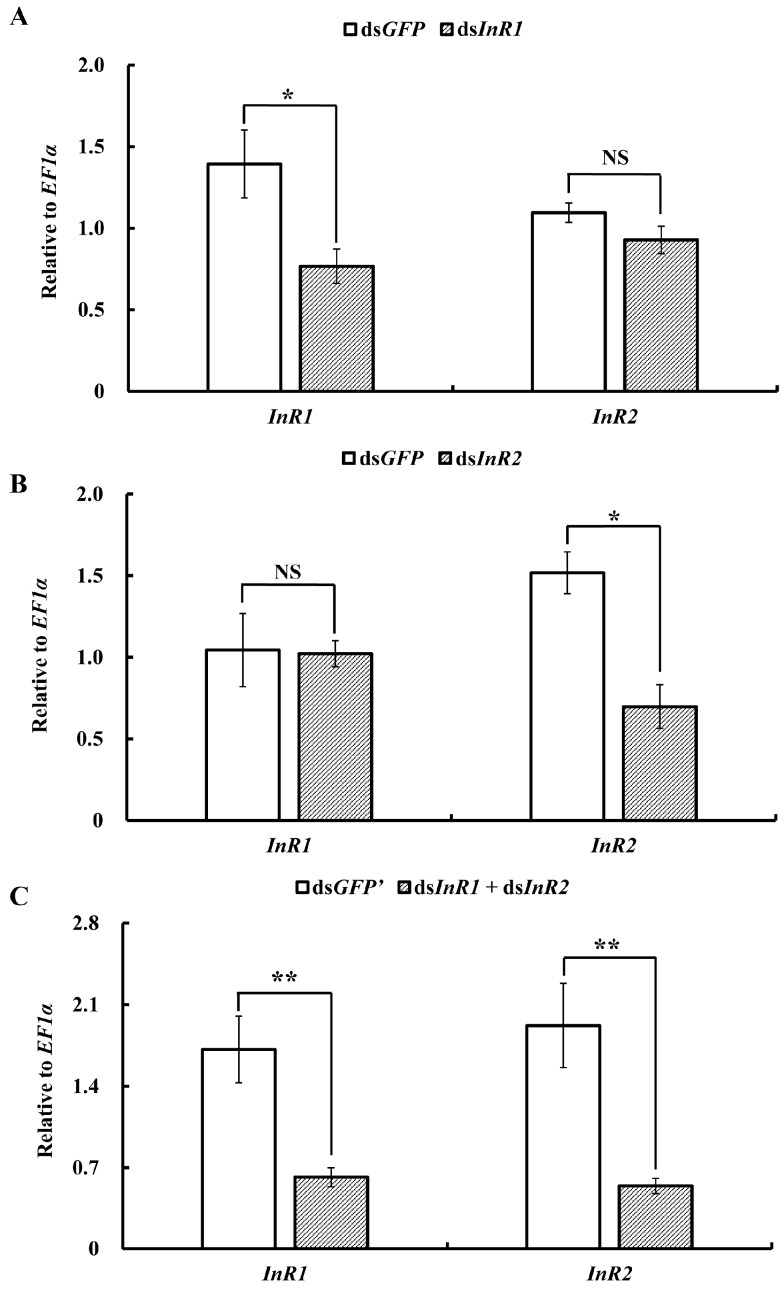
Relative expression levels of *AcInR1* and *AcInR2* after feeding on specific dsRNA. (**A**) Expression levels of *AcInR1* and *AcInR2* after feeding on ds*InR1*; (**B**) expression levels of *AcInR1* and *AcInR2* after feeding on ds*InR2*; (**C**) expression levels of *AcInR1* and *AcInR2* after feeding on a mixture of ds*InR1* and ds*InR2*. The mean (±SE) expression level is based on four biological replicates. Significant differences between treatment and control are indicated with a line with asterisks (* *p* < 0.05; ** *p* < 0.01, Student’s *t* test). “NS” indicates no significant difference between samples.

Feeding of ds*InR1* specifically silenced the expression of *AcInR1* by 45% compared to the control, while the expression of *AcInR2* was not changed (Figure 3A). Similar results were observed in the ds*InR2* treatment. The expression of *AcInR2* was reduced by 54% compared to the control, while the expression level of *AcInR1* was not changed (Figure 3B). When the aphids fed on the mixture of ds*InR1* and ds*InR2*, the expression levels of *AcInR1* and *AcInR2* were significantly down-regulated by 64% and 72%, respectively, compared to the control (Figure 3C).

**Figure 4:**
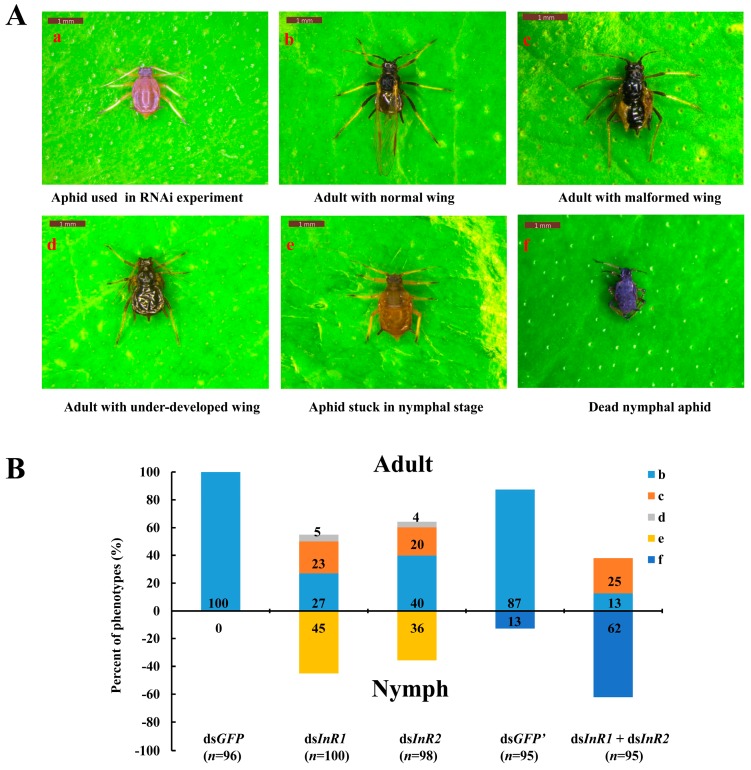
Representative phenotypes of alate *A. citricidus* after feeding on ds*InR1* or ds*InR2*, and the mixture of ds*InR1* and ds*InR2* for 72 h. (**A**) Phenotypes were presented in RNAi experiment; (**a**) Fourth instar winged-nymphs treated by dsRNA; (**b**) Adult with normal wing after RNAi; (**c**) Adult with malformed wing after RNAi; (**d**) Adult with under-developed wing after RNAi; (**e**) Aphid stuck in nymphal stage after RNAi; (**f**) Dead individuals in nymphal stages after RNAi; (**B**) the rate of presented phenotypes. “*n*” means the number of aphids in the treatment. ds*GFP* means the dsRNA concentration of the treatment was 1500 ng/μL and ds*GFP*’ means the dsRNA concentration of the treatment was 3000 ng/μL.

With effective silencing of *AcInR1* or/and *AcInR2* in fourth instar winged-nymphs (Figure 4A(a)) by dsRNA, we observed a variety of different phenotypes after treatment. These included adults with normal wings (Figure 4A(b)), adults with malformed wings (Figure 4A(c)), adults with under-developed wings (Figure 4A(d)), aphids unable to molt out of the nymphal stage (live nymphs that would die at this stage in 2–3 days) (Figure 4A(e)), and dead nymphs (Figure 4A(f)). Fisher’s exact tests of the percentage of the presented phenotypes among different treatments were performed in two ways: overall presented phenotypes and phenotypes separately. Silencing of *AcInR1* resulted in 45% of the aphids trapped in the nymphal stage, while 55% could molt from nymph to adult including 27% with normal wings, 23% with malformed wings and 5% with under-developed wings. Among aphids treated with ds*InR2*, 36% remained in the nymphal stage, 64% molted from nymphs to adults, including 40% with normal wings, 20% with malformed wings, and 4% with under-developed wings. In the control group, 100% of the aphids had normal wings. Aphids treated with the mixture of ds*InR1* and ds*InR2* were significantly different versus ds*GFP* in overall presented phenotypes (*p* = 0.000, respectively) and within each phenotype (Table 1). There was no significant difference between ds*InR1* and ds*InR2* treatments in all presented phenotypes (*p* = 0.292) as well as in the specific phenotypes (Table 1). Upon silencing of both *InR1* and *InR2*, only 38% of the aphids molted from nymphs into adults, including 13% with normal wings and 25% with malformed wings, while the rest of treated aphids (62%) died in the nymph stage. For aphids treated with ds*GFP’* (with the same dose of dsRNA as in the mixture of ds*InR1* and ds*InR2*), 87% of the aphids molted from nymphs into adults and developed with normal wings, while only 13% of the aphids were dead as nymphs (Figure 4B). Aphids treated with a mixture of ds*InR1* and ds*InR2* were significantly different versus ds*GFP*, ds*InR1*, and ds*InR2* in overall presented phenotypes (*p* = 0.000, respectively) (Table 1). For a separate analysis of phenotypes, the mixture of ds*InR1* and ds*InR2* versus ds*InR1* or ds*InR2* indicated that the mixture treatment led to more aphid mortality in nymphal stages, and fewer aphids transforming into adults, but no difference in adults with malformed wings compared to the single RNAi of *AcInR1* and *AcInR2* (*p* = 0.712 or 0.442, respectively) (Table 1).

## 3. Discussion

The genomes of three species of aphids, *A. pisum* [29], *D. noxia* [34], and *M. persicae*, were sequenced and the structure of *InRs* from *A. pisum* was predicted and characterized [2]. However, the degradation of dsRNA by feeding with an artificial diet or direct injection into aphids has suggested that aphids are insensitive to RNAi treatment [37]. Therefore, the analysis of *InRs* function in aphids using RNAi has not been carried out. Luan et al. (2013) developed a method to silence whitefly genes involved in ecdysone synthesis and signaling pathways by dsRNA feeding through a plant leaf. This resulted in reduced survival and delayed development of the nymphal stages [38]. Our previous study demonstrated that aphids also cannot molt to adults after silencing of a chitin synthase gene. Aphids have under-developed wings resulting from silencing wing-related genes through plant-stem–mediated dsRNA feeding [36,39].

This report deals with the *AcInR1* and *AcInR2* insulin receptors from *A. citricidus*. These receptors showed several important conserved features including a transmembrane segment, an intracellular tyrosine kinase (TyrKc), a furin-like cysteine-rich (Fu) region, followed by three fibronectin type 3 (FN3-1, FN3-2, and FN3-3) domains. These features are salient insulin receptor domains [40,41]. Phylogenic analyses indicated that InR and InR1 may have similar physiological functions and evolutionary relatedness in several insect groups. InR2 separates from the clade of InR1, which indicates that InR2 may play a different role compared to InR1 [6,7]. InR2 seems to be mainly present in insects with polymorphism (aphids, planthoppers, bugs, ants, bees, and dampwood termites). However, at least one non-polymorphic insect, *T. castaneum*, also has two insulin receptor genes. The relationship between the number of insulin receptors and insect species evolution needs additional work.

The activity of the ISS pathway not only depends on the expression of *InR* but also on phosphorylation events following activation by the corresponding ligands. The gene expression patterns provide information useful for predicting potential functions. *AcInR1* and *AcInR2* had different expression patterns both in alate and apterous aphids. *AcInR2* had the highest expression level in second instar nymphs and this was higher in apterous aphids than in alate aphids. The second to third instar nymph is the key period for determining the wing morph (apterous or alate) in aphids [24,42]. Our results indicate that *AcInR2* might be involved in wing dimorphism and may play a relatively more important role in apterous aphids. Similar results were found in *N. lugens*, where *NlInR2* was highly expressed in the fifth instar nymph [6] and wing dimorphism (short-wing and long-wing morphs) occurred during the fifth instar nymph to adult stages [43]. *AcInR1* had the highest expression levels in alate adults, but no difference among the nymphal stages and no difference in apterous aphids. The wing buds developed slowly in each nymphal instar until the fully formed wings unfolded after adult emergence [44]. Thus, the fourth instar nymph to adult transition was the key period of aphid wing development. Our results indicate that *AcInR1* plays an important role in development (including wing development) from fourth instar nymphs to adults. Similar differences in expression patterns of insulin receptor genes were found in the honey bee, *A. mellifera*. Both *AmInR1* and *AmInR2* had the highest expression level in eggs and different expression patterns in queens and workers, indicating that they might be associated with caste determination [18]. In *T. castaneum*, *TcInR1* is expressed at the highest levels in the old adult stage followed by the early pupal stage, whereas *TcInR2* is most highly expressed during the larval stages followed by the old adult stage, suggesting that *TcInR1* and *TcInR2* most likely perform specific functions in larval–pupal development and in reproduction at distinct developmental time points and to different extents [7].

The plant-stem–delivered dsRNA feeding experiment explored the functions of *AcInR1* and *AcInR2* and their effects on aphid development during the nymph–adult transition. *AcInR1* and *AcInR2* appear to be essential for the successful nymph-to-adult development of alate *A. citricidus*. Silencing of *AcInR1* and *AcInR2* resulted in most aphids being either unable to molt normally to the adult stage, nymphs not molting to the adult stage, or adults with deformed wings. Wing deformities (24%–28%) were common after silencing of *AcInR1* and/or *AcInR2* together with defects in the nymph–adult transition. We did not observe other aphid abnormalities following silencing of the insulin receptors. The wing deformities seen in this study may indicate that insulin receptors help to regulate wing development since the deformed aphids successfully transformed from nymphs to adults. However, advanced studies are needed to elucidate how insulin receptors could be involved in regulating wing development. No differences were seen in the rates of all presented phenotypes after ds*InR1* and ds*InR2* treatment. These findings indicate that despite the sequence differences between *InR1* and *InR2*, *InR2* shares overlapping functions with *InR1* during alate *A. citricidus* development. In the double-knockdown experiment, aphids fed the ds*InR1* and ds*InR2* mixture experienced high mortality. Silencing of *AcInR1* and *AcInR2* disrupted the development (including wing development) of the aphids during the nymph–adult transition and this might indicate that decreased capability for food intake reduced nutrient transport. Other studies have demonstrated that high expression levels of *BdInR*, *BmInR*, and *BgInR* were induced by starvation of *B. dorsalis*, *B. mori*, and *B. germanica*, respectively [3,16,45]. In *T. castaneum*, knockdown of *TcInR1* decreased food intake through the sulfakinin signal pathway in the larval stages [21]. In the present study, both *AcInR1* and *AcInR2* were involved in the nymph–adult transition and wing development. This suggests that these two insulin receptors might be conserved during aphid development, while they could also play different roles in other aphid processes. Because high mortality occurred in first instar nymphs using the RNAi system, the function of *AcInR1* and *AcInR2* in wing dimorphism needs further verification. Genome editing tools such as CRISPR/Cas9 (clustered, regularly interspaced, short palindromic repeat/CRISPR associated) [46] have been used in many insects and this tool will be helpful in exploring the distinct role of *AcInR1* and *AcInR2* in aphid wing dimorphism and the development of apterous aphids. This study focused on the nymph–adult transition and presented an approach for exploring functions of genes in aphids. More phenotypes will be examined in future work which will evaluate factors such as fecundity, lifespan, and feeding behavior.

## 4. Materials and Methods

### 4.1. Insect Culture

Alate *A. citricidus* adults were obtained in 2012 from a wild aphid population in a citrus screenhouse at Southwest University, Chongqing, China. Stock colonies were maintained on potted citrus seedlings (*Citrus sinensis*) in the laboratory at 25 ± 1 °C, 75%–80% relative humidity and 14:10 h (Light:Dark) photoperiod. Alate morphs were induced by high-density aphid rearing after transfer to fresh host plants [31,32]. All progeny were produced by parthenogenesis from the stock colony.

### 4.2. Total RNA Extraction and cDNA Synthesis

Total RNA used for gene cloning and expression levels from different developmental stages, wing morphs, and dsRNA treatment were isolated with a TRIzol kit (Invitrogen, Carlsbad, CA, USA) according to manufacturer instructions. RNA was quantified by measuring absorbance at 260 nm using a Nano Vue UV-Vis spectrophotometer (GE Healthcare Bio-Science, Uppsala, Sweden). The purity of all RNA samples was assessed from the absorbance ratio at OD260/280 and OD260/230. The RNA integrity was then checked by 1% agarose gel electrophoresis. The genomic DNA was removed by using of DNase I (Promega, Madison, WI, USA). The first strand cDNA was synthesized from 500 ng of DNA-free RNA using a PrimerScript^®^ RT Reagent Kit (Takara, Dalian, China) according to manufacturer instructions. Briefly, the 10 μL reaction system consisting of 500 ng RNA, 2 μL reverse transcription buffer, 200 pmol random 6 mers, 0.5 μL PrimerScript^®^ RT Enzyme Mix I and RNase free H_2_O. The reaction conditions included a step of 37 °C for 15 min and 85 °C for 5 s by using a C1000TM Thermal Cycler (Bio-Rad, Hercules, CA, USA). After the reverse transcription, the synthesized cDNA was stored at −20 °C for later use.

### 4.3. cDNA Cloning

Based on results of the high throughput transcriptome sequencing of *A. citricidus* (Sequence Read Archive database accession No. SRR2123649) and use of BLASTx against NCBI non-redundant (NR) protein database, we identified two unigene sequences (c13292.graph_c0 and c8883.graph_c0) that were predicted to encode the insulin receptor. The cloning strategy was designed to achieve a full-length confirmation (Table S1, InR1 and InR2). The specific PCR reactions were performed in a C1000TM Thermal Cycler and the PCR amplifications were performed in 25 μL, containing 1 μL cDNA complete, 2.5 μL 10× PCR buffer (Mg^2+^ free), 2 μL 2.5 mM Mg^2+^, 2 μL 2.5 mM dNTP Mix, 15.5 μL nuclease-free water, 1 μL of each specific primer (10 mM), and 0.25 μL rTaq^TM^ polymerase (Takara). The PCR reaction was performed as followed: an initial denaturation for 3 min at 95 °C, followed by 95 °C for 30 s, 55 to 60 °C (based on the primer annealing temperatures) for 30 s, 72 °C extension for 1 to 2 min by 35 cycles and a final extension at 72 °C for 10 min. The amplified PCR fragments were gel-purified with a Gel Extraction Mini Kit (Takara) and ligated into pGEM-T easy vector (Promega). Recombinant plasmids were sequenced subsequently by an ABI Model 3100 automated sequencer (Invitrogen Life Technologies, Shanghai, China).

### 4.4. Phylogenetic Analysis

The SMART program provided by EMBL (available on: http://smart.embl-heidelberg.de/) was used for the identification of modular domains. The transmembrane helices were analyzed using TMHMM v. 2.0 (available on: http://www.cbs.dtu.dk/services/TMHMM-2.0/). Molecular weights and isoelectric points (p*I*) of the deduced protein sequences were predicted by the COMPUTE PI/Mw program provided by ExPASy (available on: http://web.expasy.org/compute_pi/). DNAMAN 6.0 (DNAMAN 6.0, Lynnon BioSoft, Vaudreuil, QC, Canada) was used to edit the nucleotide sequences. The full-length amino acid sequences were aligned with ClustalW using MEGA 5.05. The phylogenetic trees were constructed using the maximum likelihood (ML) method with “*p*-distance” as the amino acid substitution model, “pairwise deletion” as the gaps/missing data treatment and 1000 bootstrap replications [47]. The Insulin receptor genes used to generate the tree were from 25 insects and their GenBank IDs were listed in Table S2.

### 4.5. Quantitative Reverse Transcription PCR (RT-qPCR)

To determine the expression profiles of *AcInR1* and *AcInR2* in different development stages of brown citrus aphid, thirty insects each of first, second, third, fourth instar nymphs, and 30 apterous and alate adults were collected for total RNA isolation. Alate and apterous adults were collected within 48 h after the final molt. Specific primers used for RT-qPCR analysis were designed by primer 3.0 (available on: http://bioinfo.ut.ee/primer3-0.4.0/). A RT-PCR was performed to check primer specificity before qPCR and the sequences were confirmed as described above. The qPCR was performed on a Mx3000P thermal cycler (Stratagene, La Jolla, CA, USA) with a 10 μL reaction mixture containing 0.5 μL cDNA completes, 5 μL GoTaq^®^ qPCR Master Mix (Promega), 0.5 μL of each specific primer (0.2 mM) and 3.5 μL nuclease-free water. PCR amplifications were performed with the following cycling conditions: 95 °C for 120 s, then 40 cycles of 95 °C for 30 s and 60 °C for 30 s, a final cycle of 60 °C for 30 s and 95 °C for 30 s. A standard curve was established for each primer pair with serial dilutions of cDNA (1, 1/5, 1/25, 1/125, 1/625, and 1/3125) to determine the amplification efficiencies and *C*T values. Reference gene, *EF1α*, was used to normalize the expression of genes [48] by qBase [49].

### 4.6. RNAi

RNAi was applied to explore the potential biological functions of *AcInR1* and *AcInR2* in *A. citricidus*. The primers used to synthesize dsRNA are listed in Supplementary Table S1. The TranscriptAid T7 High Yield Transcription Kit (Thermo Scientific, Wilmington, DE, USA) was using for the dsRNA synthesis. The size of the products was confirmed by electrophoresis on a 1% agarose gel and the sequences were confirmed as described above. The dsRNA concentration was 1500 ng/μL for single RNAi treatment and ds*GFP* was used as a control in the same concentration. In the co-silencing of two insulin receptors, ds*InR1* and ds*InR2* were fed simultaneously as a mixture at a 1:1 ratio, each dsRNA concentration was 3000 ng/μL and ds*GFP* concentration was 3000 ng/μL.

A RNAi method to silence gene expression level by dsRNA feeding through a citrus leaf was used based on a previous study [36]. Briefly, an 8-cm-long citrus stem with a fresh leaf was detached from the citrus seeding and inserted into a 250 μL PCR tube containing 200 μL dsRNA. Then the tube containing the dsRNA and the leaf were transferred into a 50 mL plastic tube. Twenty-five fourth-instar nymphs were released onto the leaf and representative phenotypes were observed after dsRNA treatment for 72 h.

Four biological replicates were performed for each treatment. Photos were taken using a Leica M165C microscope (Leica Microsystems, Wetzlar, Germany). To assess the down-regulation of *AcInR1* and *AcInR2* by dsRNA feeding, all surviving aphids after feeding on dsRNA for 72 h were pooled for RNA extraction to examine gene expression level and qPCR was performed as described above.

### 4.7. Statistical Analysis

The relative expression levels of *AcInR1* and *AcInR2* in different development stages were analyzed using one-way analysis of variance (ANOVA) followed by Tukey’s honestly significant difference (HSD) multiple comparison test. The level of significance was set at *p* < 0.05. The expression levels of *AcInR1* and *AcInR2* between dsRNA-treated and control were compared by using a two-tailed Student’s *t*-test at the significance levels of * *p* < 0.05 and ** *p* < 0.01. The percentage of presented phenotypes among treatments were analyzed using a two-tailed Fisher’s exact test with 2 × *n* contingency tables (* *p* < 0.05, ** *p* < 0.01, and *** *p* < 0.01). All statistical analysis was carried out using SPSS version 20.0 (IBM, Armonk, NY, USA).

## 5. Conclusions

We identified two insulin receptor genes, *AcInR1* and *AcInR2*, in the brown citrus aphid, *A. citricidus*. The gene expression patterns indicated that they play important roles in the nymph-to-adult transition. RNAi results showed that *AcInR1* and *AcInR2* are essential genes for *A. citricidus* development and have overlapping functions, even though their sequences are significantly different. The results provide a foundation for the advanced study of insulin receptors in aphids. They also provide a theoretical basis, using RNAi technology, for controlling the dispersal of this pest.

## Figures and Tables

**Table 1:** Fisher’s exact tests of presented phenotypes between different treatments.

Comparison	Presented Phenotype					
	Overall Presented Phenotypes	Adult Stage			Nymph Stage	
		Normal Wing (b in Figure 4A) versus Others	Malformed Wing (c in Figure 4A) versus Others	Underdeveloped Wing (d in Figure 4A) versus Others	Alive (e in Figure 4A) versus Others	Dead (f in Figure 4A) versus Others
ds*InR1* versus ds*GFP*	*** (*p* = 0.000)	*** (*p* = 0.000)	*** (*p* = 0.000)	* (*p* = 0.026)	*** (*p* = 0.000)	-
ds*InR2* versus ds*GFP*	*** (*p* = 0.000)	*** (*p* = 0.000)	*** (*p* = 0.000)	* (*p* = 0.045)	*** (*p* = 0.000)	-
ds*InR1* + ds*InR2* versus ds*GFP’*	*** (*p* = 0.000)	*** (*p* = 0.000)	*** (*p* = 0.000)	-	-	*** (*p* = 0.000)
ds*InR1* versus ds*InR2*	NS (*p* = 0.292)	NS (*p* = 0.056)	NS (*p* = 0.658)	NS (*p* = 0.756)	NS (*p* = 0.183)	-
ds*InR1* versus ds*InR1* + ds*InR2*	*** (*p* = 0.000)	* (*p* = 0.012)	NS (*p* = 0.712)	* (*p* = 0.027)	*** (*p* = 0.000)	*** (*p* = 0.000)
ds*InR2* versus ds*InR1* + ds*InR2*	*** (*p* = 0.000)	*** (*p* = 0.000)	NS (*p* = 0.442)	* (*p* = 0.047)	*** (*p* = 0.000)	*** (*p* = 0.000)

ds*InR1*, ds*InR2*, ds*InR1* + ds*InR2* mean that aphids feeding on ds*InR1*, ds*InR2*, and the mixture of ds*InR1* and ds*InR2*, respectively. Chi-square test: * *p* < 0.05; *** *p* < 0.001. In the test of ds*InR1* or ds*InR2* versus ds*InR1* + ds*InR2*, the Corrected mortality was used in Dead versus others.

## Supplementary Materials

Supplementary materials can be found at www.mdpi.com/1422-0067/18/2/357/s1.
