# Supplementary material for: Silencing of Two Insulin Receptor Genes Disrupts Nymph-Adult Transition of Alate Brown Citrus Aphid
Source: Int J Mol Sci. 2017 Feb 21;18(2):357. doi: 10.3390/ijms18020357 (PMC5343892; doi:10.3390/ijms18020357)
Supplement: Supplementary file 1 [file ijms-18-00357-s001.pdf]

Supplementary Materials: Silencing of Two Insulin Receptor Genes Disrupts Nymph-Adult Transition of Alate Brown Citrus Aphid

Bi-Yue Ding, Feng Shang, Qiang Zhang, Ying Xiong, Qun Yang, Jin-Zhi Niu, Guy Smagghe and Jin-Jun Wang

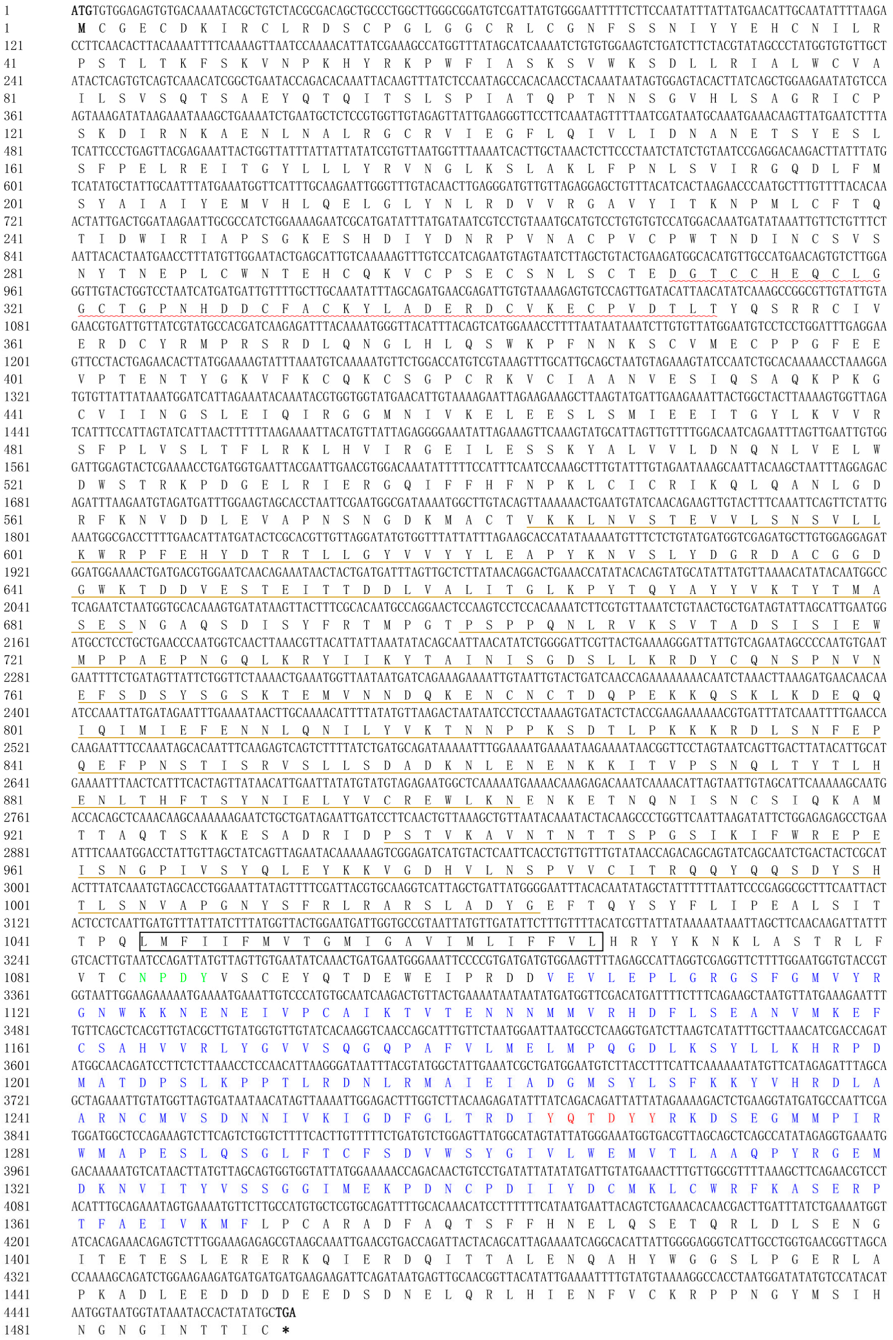

Figure S1. Nucleotide and deduced amino acid sequences of *AcInR1* from *Aphis (Toxoptera) citricida*. The start codon (ATG), stop codon (TGA) are highlighted in black. The furin-like cysteine-rich (Fu) region is underlined with red wavy lines. Three fibronectin type 3 (FN3) domains are labeled with yellow solid underlines. A single transmembrane (TM) region is highlighted with a box. An “NPXY” motif is shown in green. The highly conserved tyrosine kinase domain (TyrKc) is indicated in blue. A triple tyrosine cluster (YXXXYY) is indicated in red.

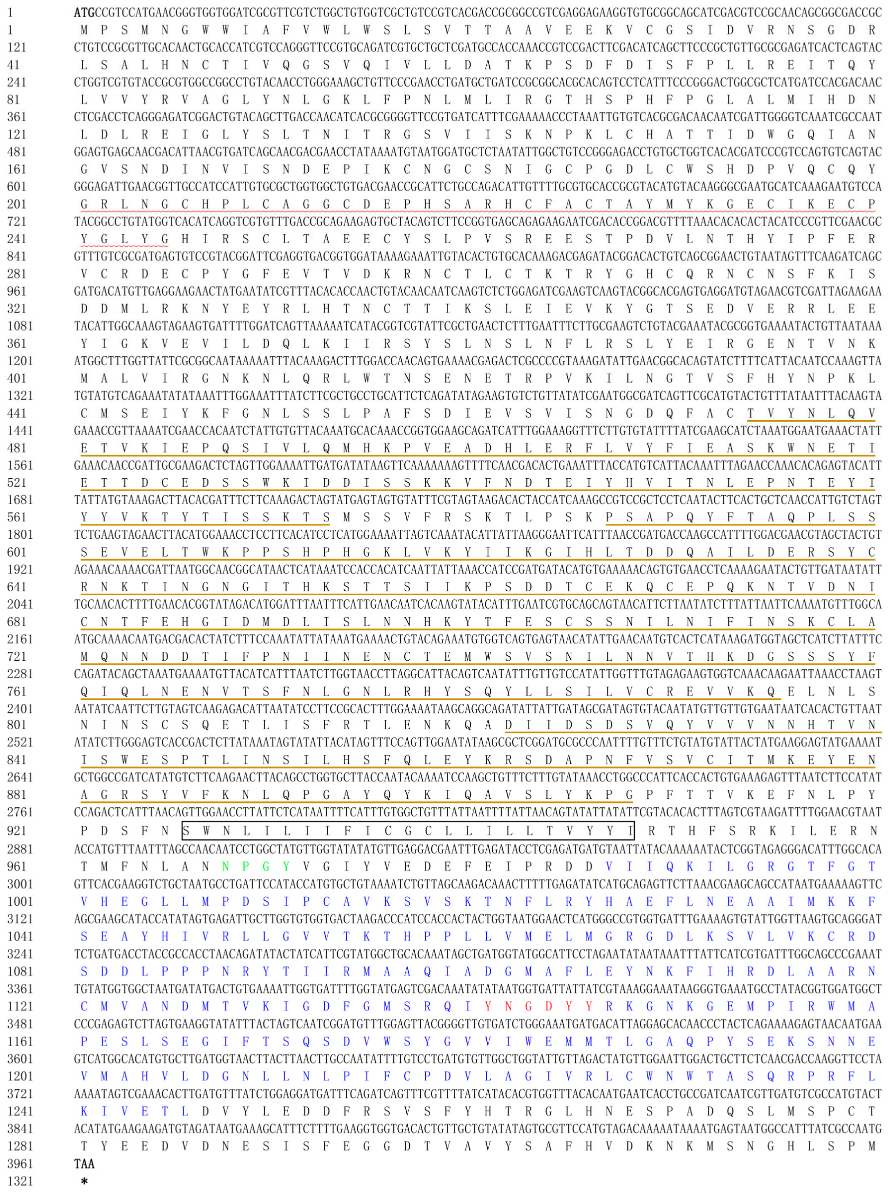

Figure S2. Nucleotide and deduced amino acid sequences of *AcInR2* from *Aphis (Toxoptera) citricida*. The start codon (ATG), stop codon (TGA) are highlighted in black. The furin-like cysteine-rich (Fu) region is underlined with red wavy lines. Three fibronectin type 3 (FN3) domains are labeled with yellow solid underlines. A single transmembrane (TM) region is highlighted with a box. An "NPXY" motif is shown in green. The highly conserved tyrosine kinase domain (TyrKc) is indicated in blue. A triple tyrosine cluster (YXXXYY) is indicated in red.

Table S1. Primer sequences used for cloning, quantitative real-time PCR and double-stranded RNA (dsRNA) synthesis.

Experiments	Primer Names and Sequence (5′ to 3′)	Amplification Efficiency	Product Length
Full-length confirmation	*InR1*-F: ATGTGTGGAGAGTGTGACAA<br>*InR1*-R: TCAGCATATAGTGGTATTTAT	-	4473
	InR2-F: ATGCCGTCCATGAACGGGTG<br>*InR2*-R: TTACATTGGCGATAAATGGCC	-	3963
qPCR analysis	Q-*InR1*-F: ATGGTCGAGATGCTTGTGGA<br>Q-*InR1*-R: TGTGGAGGACTTGGAGTTCC	98.2%	217
	Q-*InR2*-F: TTCCCGAACCTGATGCTGAT<br>Q-*InR2*-R: GCTCACTCCATTGGCGATTT	96.3%	204
	Q-*EF1α*-F: GATGCACCTGGTCACAGAGA<br>Q-*EF1α*-R: CCATCTTGTTCACACCAACG	96.5%	194
dsRNA synthesis	ds*AcInR1*-F: taatacgactcactatagggTTGTGGGATTGGAGTACTC<br>ds*AcInR1*-R: taatacgactcactatagggCACTTTGTGCACCATTAGA	-	511
	ds*AcInR2*-F: taatacgactcactatagggAACCCTAAATTGTGTCACG<br>ds*AcInR2*-R: taatacgactcactatagggGACTTCGATCTCCAGAGAC	-	609
	ds*GFP*-F: taatacgactcactatagggCAGTTCTTGTTGAATTAGATG<br>ds*GFP*-R: taatacgactcactatagggTTTGGTTTGTCTCCCATGATG	-	436

InR, insulin receptor; dsRNA, double-stranded RNA; *EF1α, elongation factor 1α*; F, forward; R, reverse; Ac, *Aphis (Toxoptera) citricida*. The lower case letters represent the T7 promoter sequences for efficient in vitro transcription in dsRNA synthesis.

Table S2. Sequences and relevant information used for phylogenetic analysis of the insulin receptor genes.

Genes	GenBank No. or Gene ID	Species
BgInR	CDI30232.1	*Blattella germanica*
ZnInR1	KDR13786.1	*Zootermopsis nevadensis*
ClInR1	XP_014256336.1	*Cimex lectularius*
HhInR1	XP_014273515.1	*Halyomorpha halys*
NlInR1	AIY24638.1	*Nilaparvata lugens*
AcInR1	KX507134	*Aphis (Toxoptera) citricida*
DnInR1	XP_015375915.1	*Diuraphis noxia*
ApInR1	XP_008185917.1	*Acyrthosiphon pisum*
MpInR1	MYZPE13164_G006_v1.0_000181710	*Myzus persicae*
PhInR	XP_002430961.1	*Pediculus humanus corporis*
NvInR	XP_003425750.1	*Nasonia vitripennis*
AmInR1	NP_001233596.1	*Apis mellifera*
BiInR1	XP_003490625.1	*Bombus impatiens*
SiInR1	XP_011158641.1	*Solenopsis invicta*
CfInR1	XP_011251746.1	*Camponotus floridanus*
HsaInR1	XP_011144146.1	*Harpegnathos saltator*
OnInR	AFQ20827.1	*Onthophagus nigriventris*
TcInR1	XP_008199415.1	*Tribolium castaneum*
BmInR	XP_012549124.1	*Bombyx mori*
PxInR	XP_011567916.1	*Plutella xylostella*
AaInR	AAB17094.1	*Aedes aegypti*
DmInR	ACY01695.1	*Drosophila melanogaster*
GmInR	AFQ01096.1	*Glossina morsitans morsitans*
BdInR	XP_011210333.1	*Bactrocera dorsalis*
CcInR	XP_004518075.1	*Ceratitis capitata*
CfInR2	XP_011263722.1	*Camponotus floridanus*

Table S2. *Cont.*

Genes	GenBank No. or Gene ID	Species
SiInR2	XP_011175961.1	*Solenopsis invicta*
HsaInR2	XP_011140707.1	*Harpegnathos saltator*
AmInR2	XP_394771.4	*Apis mellifera*
BiInR2	XP_003484422.1	*Bombus impatiens*
ZnInR2	KDR21367.1	*Zootermopsis nevadensis*
ClInR2	XP_014242610.1	*Cimex lectularius*
HhInR2	XP_014273071.1	*Halyomorpha halys*
NlInR2	AIY24639.1	*Nilaparvata lugens*
TcInR2	AHF20215.1	*Tribolium castaneum*
AcInR2	KX507135	*Aphis (Toxoptera) citricida*
DnInR2	XP_015363980.1	*Diuraphis noxia*
ApInR2	XP_001942660.2	*Acyrthosiphon pisum*
MpInR2	MYZPE13164_G006_v1.0_000187240	*Myzus persicae*
HsInR	NP_001073285.1	*Homo sapiens*
